# Tonic Dopamine Sensing Reveals a D2/D3 Mediated Dopamine Response to Raclopride in ClockΔ19 Mice Model

**DOI:** 10.21203/rs.3.rs-7403119/v1

**Published:** 2025-09-18

**Authors:** Bingchen Wu, Elisa Castagnola, Elaine Robbins, Mariya Kaminsky, Subramaniam Sanker, Colleen McClung, Xinyan Tracy Cui

**Affiliations:** University of Pittsburgh; Louisiana Tech University; University of Pittsburgh; University of Pittsburgh; University of Pittsburgh; University of Pittsburgh; University of Pittsburgh

## Abstract

The circadian rhythm regulates physiological and behavioral processes, with disruptions linked to metabolic and neuropsychiatric disorders. Circadian genes play a crucial role in the regulation of dopaminergic signaling, yet the underlying molecular mechanisms remain unclear. This study investigates how the *Clock* gene modulates dopamine (DA) dynamics using *in vivo* electrochemical DA sensing and molecular profiling. Utilizing carbon fiber electrodes (CFEs) with poly(3,4-ethylenedioxythiophene)/carbon nanotube (PEDOT/CNT) coatings, we measured extracellular DA levels in the striatum of wild-type (WT) and *Clock*Δ19 mutant mice via square wave voltammetry (SWV). Pharmacological perturbation with raclopride (D2/D3 receptor antagonist) and nomifensine (dopamine reuptake inhibitor) revealed an increased DA receptor sensitivity in *Clock*Δ19 mice, with a significantly faster DA response to raclopride. Molecular profiling via qRT-PCR showed elevated *tyrosine hydroxylase* (*TH*) expression in the ventral tegmental area (VTA) of *Clock*Δ19 mice, suggesting increased DA synthesis. Additionally, *Clock*Δ19 mice exhibited higher expression of D2 and D3 dopamine receptors and *glutamate decarboxylase 67* (*Gad67*) in the VTA, implicating altered dopaminergic and γ-aminobutyric acid (GABA)ergic regulation. These findings highlight the *Clock* gene’s role in DA homeostasis, revealing its impact on neurotransmission.

## Introduction

The circadian rhythm—an endogenous cycle regulating behavioral and physiological changes—enables organisms to adapt to environmental fluctuations and optimize survival strategies. These rhythmic activities are synchronized by external cues known as zeitgebers (ZTs), such as light and food [1, 2]. The interplay between neural and molecular clock mechanisms ensures the stability of these rhythms. In mammals, the suprachiasmatic nucleus (SCN) serves as the central pacemaker, coordinating rhythmic activity across various brain regions and peripheral tissues, which may function either independently or under SCN regulation [3].

One of the core transcription factors that affect the diurnal regulation of the mammalian molecular circadian rhythms is the *circadian locomotor output cycles kaput* (*Clock)* gene [1, 4, 5]. The molecular clock machinery, present in nearly all cell types, operates through a series of transcriptional-translational feedback loops to regulate rhythmic processes [5]. Disruptions to this delicate rhythm, often caused by jet lag, shift work, or exposure to artificial light at night, can significantly affect health and well-being. Such disruptions are associated with an increased risk of various chronic diseases, including diabetes, cardiovascular disease, and depression [6].

Emerging evidence suggests that circadian genes play a crucial role in dopamine signaling [1, 7–14]. Disrupting the *Clock* gene globally increases the firing and bursting activity of VTA dopaminergic neurons, which project to the striatum and release DA [7]. Moreover, knocking down expression of *Clock* specifically in the VTA region recapitulates this effect, suggesting that the *Clock* influences dopaminergic activity locally within dopamine neurons [15].

The association between striatum DA dynamics and circadian rhythm has garnered significant attention. The striatum, a critical region for motor control, cognitive processing, reward mechanisms, decision-making, and emotional regulation, is predominantly composed of GABAergic medium spiny neurons (MSNs) expressing dopamine receptors [16, 17]. DA levels within the striatum exhibit diurnal fluctuations [1, 8–10, 18–21], and chronic direct DA sensing has demonstrated that the *Clock* gene mutation influences extracellular DA levels [12]. At the molecular level, dopamine receptors modulate a wide range of physiological functions [22, 23]. Specifically, D2/D3 receptor expression has been strongly linked to both circadian rhythm regulation and reward processing [16, 17].

*Clock*Δ19 mutant mice have been used as a primary model to study the effects of *Clock* gene disruption on dopaminergic transmissions. These mice have a dominant-negative mutation in the *Clock* gene and have been shown to exhibit increased cocaine sensitivity and preference, increased locomotor activity, reduced anxiety- and depression-like behavior, increased intracranial self-stimulation at a lower threshold, and increased dopaminergic cell activity in the VTA [7, 14, 24–26]. Circadian genes have also been shown to directly regulate the expression of the dopamine receptors in striatal regions [27] [28]. Moreover, normal molecular rhythms in these receptors are disrupted with excess dopamine caused by exposure to chronic cocaine [28]. However, the molecular mechanisms that link dopamine signaling dynamics to the *Clock* gene expression remain unclear.

**Figure 1:**
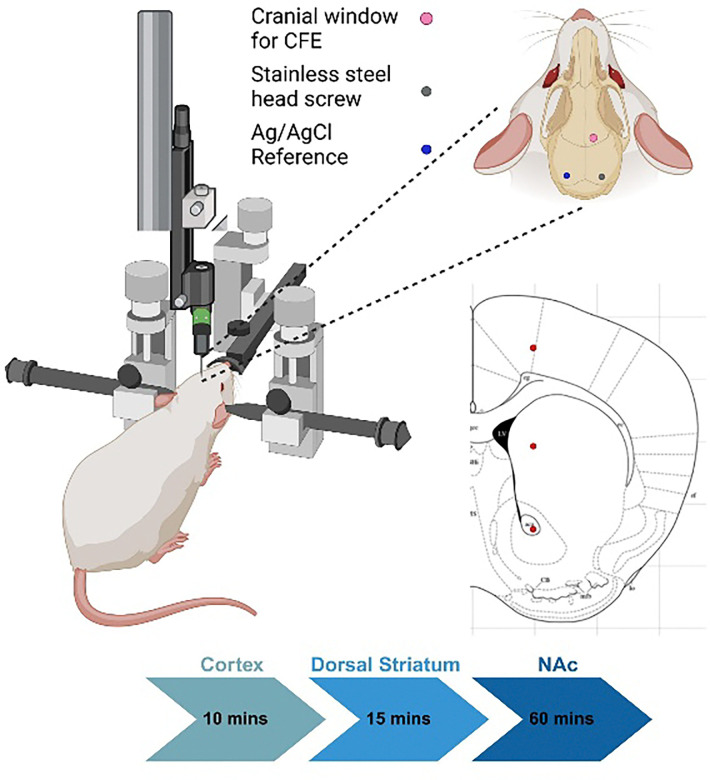
*In vivo* experimental set-ups. Mice were anesthetized and head-fixed using a stereotaxic frame. A CFE was implanted in the motor cortex, a stainless-steel screw was placed over the ipsilateral visual cortex as counter electrode, and an Ag/AgCl reference electrode with a salt bridge was placed over the contralateral side. The CFE was advanced from the cortex to the dorsal striatum and eventually reached NAc regions (4.5 mm).

In this study, we employed direct electrochemical DA sensing combined with molecular profiling techniques to investigate how the *Clock* gene regulates DA dynamics at both the system and molecular levels. Our previous work has demonstrated robust *in vivo* tonic DA sensing using carbon fiber electrodes (CFEs) coated with poly(3,4-ethylenedioxythiophene) doped with acid-functionalized carbon nanotubes (PEDOT/CNT) in rat models, using square wave voltammetry (SWV) [12, 29]. with the same technique, we performed *in vivo* DA measurements in the striatum of wildtype (WT) and *Clock*Δ19 mutant mice (Fig. 1). To further investigate DA circuitry, we administered raclopride (a D2/D3 receptor antagonist) and nomifensine (a dopamine reuptake inhibitor) to both groups and monitored DA responses using CFEs. Additionally, quantitative real-time PCR (qRT-PCR) analysis was conducted to compare the expression levels of key DA receptors in the VTA and nucleus accumbens (NAc) between WT and *Clock*Δ19 mice. By combining real-time extracellular DA measurements with molecular profiling, this study provides novel insights into the role of the *Clock* gene in regulating dopaminergic circuits.

## Results

### Electrochemical Performance of CFEs and Dopamine Measurements in WT and ClockΔ19 Mice

**Figure 2:**
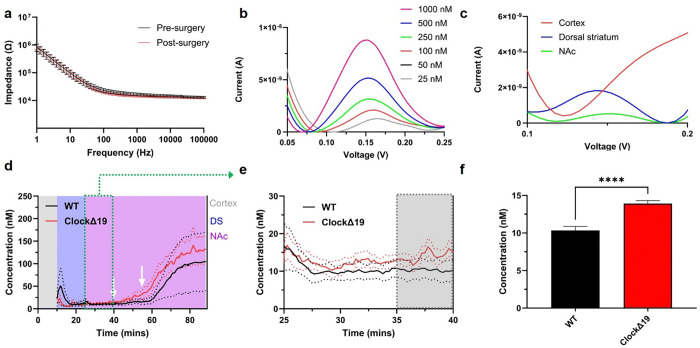
CFEs characterization. **a),** EIS comparison before implantation and after explanting. n = 10. **b),** Representative SWV measurements for post-calibration of explanted CFEs **c),** Representative SWV when CFEs are in different brain regions in vivo. **d),** Average DA dynamics during the 85 min experimental window. White arrows indicate the timing of injections: Raclopride at 40 mins and nomifensine at 55 mins. Dotted lines are standard error of the mean (SEM) for each group. **e)**, Zoomed in temporal dynamics of baseline DA in NAc region of WT and *ClockΔ19* mice (Green box portion from **d**). The gray box indicates the 5-min time bin used for quantifying the average DA response shown in f). **f),** Quantitative DA concentration comparison in NAc region of WT and *ClockΔ19*mice. *ClockΔ19* has significantly higher DA in the NAc than WT.Welch’s t test, **** p<0.0001. (5 animals in WT, 6 animals in *ClockΔ19* group, mean ± SEM)

The electrochemical performance of PEDOT/CNT coated CFEs was first evaluated to assess their stability and reliability as DA sensors. Electrochemical impedance spectroscopy (EIS) measurements taken before implantation and after explantation from mouse brains exhibited minimal changes, indicating that the PEDOT/CNT coating on CFEs is both mechanically and electrochemically stable (Fig. 2a). To confirm electrode sensitivity post-implantation, a calibration was performed on the explanted CFEs. The SWV calibration waveforms demonstrated that the electrodes remained responsive to DA (Fig. 2d). The observed shifts in DA redox peak potential could be attributed to biofouling, differences in ionic strength, or pH variations between *in vivo* and *in vitro* conditions.

During the *in vivo* testing, CFE was first placed in the cortex for 10 min, then advanced to DS for 15 min and eventually advanced to the NAc. Representative SWV measurements from different brain regions revealed varying levels of DA levels (Fig. 2c). No detectable DA signals were observed when CFEs were positioned in the cortex, which is consistent with the known low DA level in the motor cortex. In contrast, a well-defined DA redox peak at approximately 0.15 V was evident in the DS and the NAc regions (Fig. 2c). To facilitate direct comparison, DA current values were converted to DA concentrations using pre-calibration from previous work [29].

The DA dynamics over the whole experimental window (90 mins) is shown in Fig. 2d. Both groups exhibited an increase in extracellular DA levels immediately following CFE implantation into the DS (Fig. 2d). This initial increase can be attributed to cellular damage caused by electrode insertion, which ruptured cells and released intracellular DA into the extracellular space. The CFE was then advanced into the NAc region, and the baseline response was tracked for 15 minutes, followed by the pharmacological manipulations. Both the WT and *Clock*Δ19 groups showed elevated DA concentrations after the raclopride (at 40mins) and nomifensine (at 55 mins) injections (Fig. 2d). The average DA concentration was calculated using the last 5 mins of each segment of the experimental window and compared between groups. *Clock*Δ19 had significantly higher levels of DA in the NAc region compared to WT (Fig. 2e&f).

### Differential DA Response to Raclopride in WT and ClockΔ19 Mice

**Figure 3:**
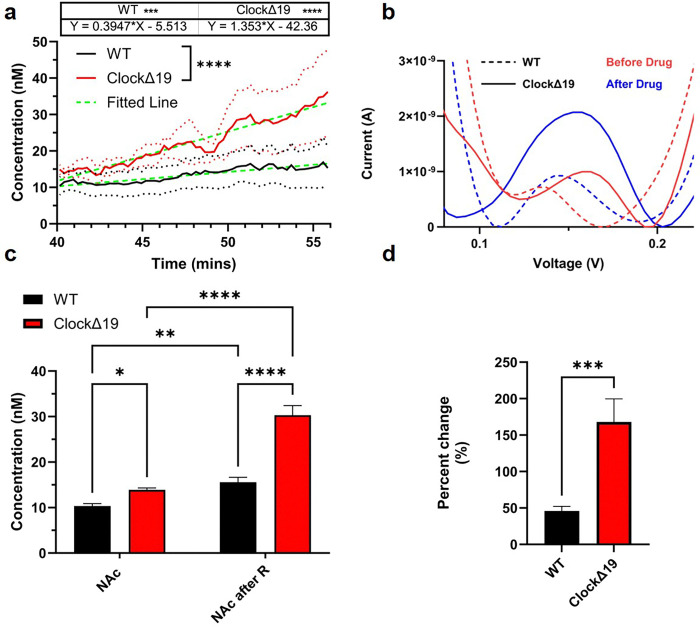
Drug-stimulated DA level comparison between WT and *ClockΔ19* mice in NAc after raclopride injection. Raclopride was injected at 40 mins. **a),**DA dynamics 15 mins after raclopride injection. Simple linear regression showed that both WT and *ClockΔ19* had significantly elevated responses after raclopride injections. *ClockΔ19* had a significantly faster response to raclopride compared with WT. 5 animals each group, simple linear regression. *** p< 0.001, ****p<0.0001. **b),**Representative SWV waveform before and after raclopride injections. **c),**The last 5 mins of DA level for each segment were averaged and compared for WT and *ClockΔ19*. Both groups showed significantly elevated DA levels DA levels after the injections of raclopride compared to baseline. *ClockΔ19* also had significantly higher baseline DA levels and raclopride induced DA increase in the NAc than WT. 5 animals in WT, 6 animals in *ClockΔ19* group, Mean ± SEM. 2-way ANOVA, Fisher’s LSD. * p<0.05, ** p< 0.01, ****p<0.0001. **d),** Percent change is calculated with the delta DA level before and after injection divided by the baseline level. *ClockΔ19* showed a significantly larger response compared to WT. 5 animals in WT, 6 animals in *ClockΔ19* group. Welch t-test. All data shown is Mean ± SEM. ***p<0.001.

After CFEs were advanced into the NAc region, the DA system was pharmacologically modulated with two types of drugs: raclopride (a D2/D3 receptor antagonist) and nomifensine (a dopamine reuptake inhibitor). To examine the effects of individual drugs, we separated different portions of the DA dynamics for analysis. For the first segment, looking at the effects of raclopride alone, the baseline DA levels recorded between 35–40 minutes and the post-raclopride levels recorded between 50–55 minutes were quantified and compared (Fig. 3). We observed an interesting differential response between the WT and the *Clock*Δ19 group. Both groups exhibited significantly positive slopes, confirming that DA levels increased following raclopride administration.

However, the *Clock*Δ19 group exhibited a significantly steeper slope compared to the WT group, indicating a more rapid and pronounced response to raclopride (Fig. 3a). Representative SWV waveforms for both WT and *Clock*Δ19 mice before and after raclopride injection are shown in Fig. 3b. Prior to raclopride administration, both groups displayed DA redox peaks at approximately 0.15 V. Following raclopride injection, both groups showed an increase in DA concentration, as evidenced by larger redox peak currents. Notably, the *Clock*Δ19 group demonstrated a more substantial increase in peak current relative to WT mice (Fig. 3b). Quantification of average DA levels during the final five minutes of each segment revealed significantly elevated DA concentrations in both groups after raclopride treatment compared to baseline (Fig. 3c). Importantly, the *Clock*Δ19 group exhibited a significantly greater increase in DA levels than WT mice. (Fig. 3c). To further assess the differential effects of raclopride in the *Clock*Δ19 group, the percentage change in DA concentration (relative to baseline) was calculated. The *Clock*Δ19 group exhibited a significantly greater percent increase in DA concentration post-raclopride compared to the WT mice. This indicates a more sensitive response of the *Clock*Δ19 group to raclopride.

**Figure 4:**
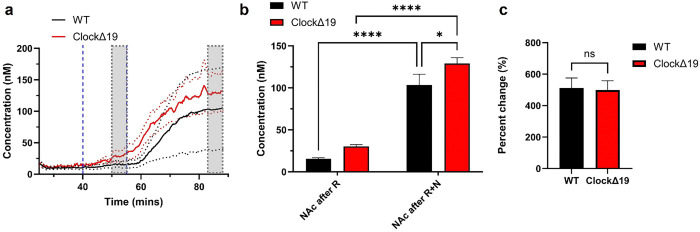
Tonic DA dynamics comparison after raclopride and nomifensine injection in WT and *ClockΔ*19. **a**, DA response over 60 mins of experimental window. Blue lines indicate injections time. Gray boxes indicate 5-minute time bin used for quantification. **b**, 5-min bin average DA concentration comparison of WT and *ClockΔ*19 after raclopride injections and raclopride + nomifensine. Both groups showed significantly increased DA levels after nomifensine injection. *ClockΔ*19 showed significantly higher DA after nomifensine injection than WT. 5 animals in WT, 6 animals in *ClockΔ*19 group, Mean ± SEM. 2-way ANOVA, Bonferroni’s multiple comparisons test. * p< 0.05, **** p<0.0001. **c**, Percent change is calculated with the delta DA level after nomifensine and after raclopride injection divided by the after raclopride DA level. *ClockΔ*19 had similar response level compared to WT. Welch t-test. All data shown is Mean ± SEM.

Next, the effect of nomifensine, a dopamine reuptake inhibitor, was examined and compared between WT and *ClockΔ19* mice (Fig. 4). A rapid increase in DA concentration was observed approximately 5 minutes after nomifensine administration at the 30-minute mark in both groups, with levels plateauing around 60-minute mark (Fig. 4a). Both WT and *Clock*Δ19 groups showed significantly elevated DA levels following nomifensine injection compared to the post-raclopride phase. The *Clock*Δ19 group continued to display significantly higher absolute DA levels than WT mice after nomifensine treatment (Fig. 4b). Interestingly, the percentage increase in DA levels from the post-raclopride baseline was similar between the two groups, indicating a comparable sensitivity to nomifensine (Fig. 4c).

### Gene Expression Analysis in the VTA and NAc

**Figure 5:**
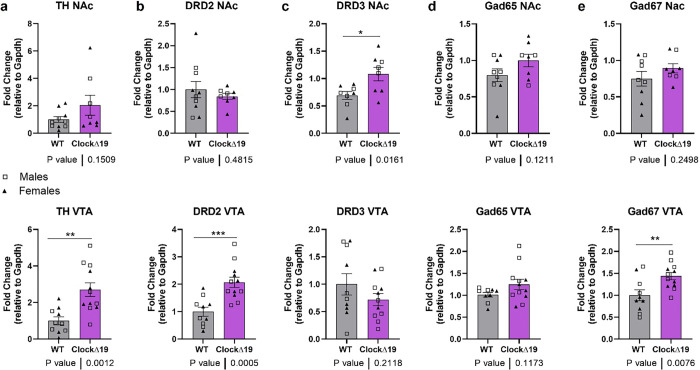
The expression level of relevant genes that are involved in DA dynamic regulations in both the VTA and NAc regions. **a)**, Comparison of *TH*expression level in the NAc and VTA regions between WT and *ClockΔ*19. Both groups showed similar expression levels in the NAc, but *ClockΔ*19 had significantly higher expression levels of *TH* in the VTA regions compared to WT. **b)**, *ClockΔ*19 showed similar dopamine receptor D2 (DRD2) expression levels in the NAc compared with WT, but significantly higher expression levels in the VTA. **c)**, *ClockΔ*19 showed significantly higher dopamine receptor D3 (DRD3) expression levels in NAc compared with WT, but similar expression levels in the VTA. **d)**, No difference was observed in *Gad65* expression between two groups in both NAc and VTA regions. **e)**, Significantly higher expression of *Gad67*from *ClockΔ*19 was observed in the VTA, while WT and *ClockΔ*19 had similar *Gad67* expression in the NAc. Unpaired T-test. All data shown is Mean ± SEM.

To explore potential molecular underpinnings of these differences, quantitative qRT-PCR was used to assess the expression of key genes involved in DA regulation in the VTA, the location of the dopaminergic neuronal soma that project to NAc, and NAc, where the terminals of those DA neurons are located. (Fig. 5). *Tyrosine hydroxylase* (*TH*), the rate-limiting enzyme in DA synthesis, exhibited comparable expression levels between groups in the NAc but was significantly upregulated in the VTA of *Clock*Δ19 mice, consistent with prior studies (Fig. 5a) [7]. Interestingly, D2 expression was similar between genotypes in the NAc but significantly elevated in the VTA of *Clock*Δ19 mice (Fig. 5b). This suggests brain region specific differences in dopamine receptor expression caused by the *Clock* gene disruption. D3 expression was significantly higher in the NAc of *Clock*Δ19 mice, whereas VTA expression remained comparable between groups (Fig. 5c).

GABA plays a crucial role in modulating DA activity in both the VTA and NAc regions [30, 31]. Glutamate decarboxylase (*Gad65* and *Gad67*) are the two isoforms of GAD responsible for GABA synthesis. *Gad65* is mainly involved in GABA vesicular release, while *Gad67* maintains basal GABA levels [32–35]. *Gad65* expression levels are similar in both NAc and VTA regions between the WT and the *Clock*Δ19 (Fig. 5d). In contrast, the *Clock*Δ19 group showed significantly elevated *Gad67* expression levels in the VTA than the WT. Both groups share similar levels in the NAc.

## Discussion

### Direct probing of DA dynamics in the NAc region

The NAc is well-known for its role in reward processing and motivation [36–40]. Previous research has shown that increased dopaminergic transmission in *Clock*Δ19 mice is linked with manic and addiction-like behaviors [27, 28]. Using our custom designed CFEs, here we find that *Clock*Δ19 mice have abnormally high levels of DA in the NAc region compared to WT mice(Fig. 2e&f). This result directly demonstrates that disrupting the *Clock* gene results in abnormally high DA levels in the NAc. Moreover, the *Clock*Δ19 mice have a significantly greater percent increase in DA concentration post-raclopride compared to the WT mice, indicating a more sensitive response of the *Clock*Δ19 group to raclopride. This result also corroborates previous studies showing that *Clock*Δ19 mice exhibit stronger behavioral effects of DA transporter blockade via raclopride administration compared to wild type mice [7, 27].

Nomifensine binds to dopamine transporters (DAT) to block DA reuptake. An increase in striatal measures of dopamine metabolites homovanillic acid (HVA) and 3,4-dihydroxyphenylacetic acid (DOPAC) in *Clock*Δ19 mice compared to wild type was also reported previously, suggesting that there is increased dopamine release and turnover [27]. However, the similar magnitude of response between WT and *Clock*Δ19 towards nomifensine in our study indicates that *ClockΔ19* mutation does not affect DAT function. On the other hand, raclopride specifically interacts with D2/D3 receptors and the more pronounced response of the *ClockΔ19* group to raclopride suggest that there might be alterations in D2/D3 receptor activity. These results provide evidence that the *Clock* gene modulates receptor-specific regulation of dopamine dynamics. The *Clock* gene mutation could influence dopamine receptor sensitivity and the downstream signaling magnitude that results in DA dynamic anomalies. Previous studies have established some connections between DA receptors and circadian genes [11, 41]. For example, rhythmic expression of the circadian protein PER2 in the dorsal striatum depends on daily dopaminergic activation of D2 receptors [10]. Loss of the *Clock* gene function also results in D1 and D2 receptor protein expression level and ratio changes [27]. In our study, the observed extracellular DA responses in *Clock*Δ19 mice warrant further investigation into the underlying molecular mechanisms.

### Gene Expression Anomalies in the VTA and NAc.

Previous studies have shown that though D2 heteroreceptors located on medium spiny neurons participate somewhat in the DA level regulation, control of DA release is mostly provided by D2 auto-receptors on dopaminergic neurons in the VTA. The loss of D2 auto-receptors in the VTA disrupts prominent feedback mechanisms regulating DA release [42–44]. In addition to D2, D3 receptors play a significant role in drug addiction, reward processing, and neuroinflammation regulation [45–49]. Similar to D2, as a member of the D2-like receptors, D3 also mediates inhibitory neurotransmission of MSNs [23, 45, 48, 50]. The *Clock* gene has been reported to regulate D3 receptor expression and tune D3 receptors’ sensitivity towards DA, forming feedback between circadian regulators and D3 receptor signaling [51, 52]. There’s also a report of strong reduction in the locomotor response and downstream signaling response in the NAc to a dopamine D1 receptor agonist in *Clock*D19 mice, suggesting an attempt to compensate for the high extracellular levels of dopamine in striatal regions [27]. On top of the D1 results, the *Clock*Δ19 group’s significantly elevated *Gad67* expression levels in the VTA region (upstream of the NAc) suggests alterations in basal GABAergic tone and increased GABAergic activity may also be compensatory to the abnormally high DA level in the NAc and VTA. Taken together, these results suggest a complex interaction between the *Clock* gene and overall dopaminergic transmission between the VTA and NAc which impacts multiple receptors and cell types, resulting in abnormal behavior.

## Conclusion

This study highlights the electrochemical stability of CFEs for DA detection and reveals significant differences in DA dynamics between WT and *Clock*Δ19 mice. The *Clock* gene mutation appears to enhance DA sensitivity to D2/D3 receptor antagonist, as evidenced by a more rapid and pronounced response to raclopride in the NAc of *Clock*Δ19 mice. qRT-PCR findings suggest that these alterations may be driven by differences in DA synthesis (TH), receptor expression (D2, D3), and there may be compensatory changes in GABAergic regulation (GAD67). Previous extracellular DA measurement was primarily done by using homogenized tissue, which can provide a measurement of the DA levels that both the intracellular and extracellular DA, and the temporal resolution is poor. By using the SWV with PEDOT/CNT coated CFE, we can directly measure extracellular DA concentration and provide higher accuracy and more dynamic information. Our findings provide new insights into the interplay between circadian genes and extracellular DA levels, and provided the bases for future studies investigating the molecular mechanisms underlying these effects as well as their behavioral manifestation

## Experimental methods

### Materials

3,4-ethylenedioxythiophene (EDOT), Raclopride, Nomifensine, nitric acid (95% fuming), and sulfuric acid were purchased from Sigma-Aldrich (St. Louis, MO, USA). Muti-wall carbon nanotubes (CNT) (10–20nm diameter, 10–30μm length, 95%, 200–350 m^2^/g) were purchased from Cheap Tube (Grafton, VT, USA).

### Electrochemistry Methods

All electrochemical procedures were conducted using a three-electrode set-up (working electrode: individual CFE electrodes; reference electrode: Ag/AgCl; counter electrode: Pt (*in vitro*)/stainless-steel bone screw (*in vivo*). An Autolab potentiostat/galvanostat, PGSTAT128N (Metrohm, Herisau, Switzerland) was used for all square wave voltammetry (SWV) procedures and electrochemical Impedance spectroscopy (EIS) measurements (*in vivo* and *in vitro*). SWV potential was swept from scanned − 0.2v to 0.3V using a 25 Hz pulse frequency, 50 mV pulse amplitude, and 5 mV step height. A liner scan from 0.3V to 0V at 1v/s was applied after the SWV waveform. The potential was held at 0V between scans.

### CNT functionalization and PEDOT/CNT deposition.

CNTs were functionalized following previous methods [53, 54]. In brief, 200 mg of multiwalled carbon nanotubes into 25 ml of concentrated nitric acid and 75 ml of concentrated sulfuric acid. This solution was sonicated for 2 hr, and then stirred overnight at 35°C. The solution was dialyzed in a DI water bath until the solution became pH neutral. The water bath was changed every 12 hr. Samples were vacuum dried and stored at 4°C.

For PEDOT/CNT deposition, 1 mg/mL of functionalized CNTs was resuspended in DI H_2_O by sonication for 10 mins. EDOT was added to this solution to a concentration of 0.01 M. The solution was then sonicated for 10 mins using a Q500 probe sonicator (1s on, 2s off, 30% power) (Qsonica L.L.C, Newtown, CT, USA). Electrochemical deposition was performed using chrono-coulometry. The applied voltage is 0.9V, with a charge cut off at 150mC/cm^2^.

### Animal housing and breeding.

Mice were housed on a 12/12 light/dark cycle (lights on 7 a.m., lights off 7 p.m.) with food and water ad libitum. *Clock*D19 mice on a Balb/c mixed background were bred as heterozygotes to produce WT and homozygous MU littermates. Female *Clock* mutant (*Clk*Δ19/*Clk*Δ19) and wild-type (+/+) littermate controls, 15–19 weeks old, were used in all studies.

### Surgical procedure

All animal work was performed under the guidelines of the University of Pittsburgh Institutional Animal Care and Use Committee (IACUC). The approved protocol ID is 22109970. Mice were anesthetized under isoflurane (2.5%) and head-fixed in a stereotaxic frame (David Kopf Instruments, Tujunga, CA, USA). Animal body temperature was maintained at 37°C using an isothermal pad connected to a SomnoSuite system (Kent Scientific Corporation, Torrington, CT, USA). Heart rates were monitored using SomnoSuite system as well. A holder was used to secure CFEs. One skull screw was carefully positioned above the left visual cortex of the mice. A 0.7mm diameter window above the ventral striatum of the right and left hemispheres was opened using a motorized drill. The coordinates for the center of the window were 1 mm posterior to Bregma and 1.1 mm lateral to the midline. The Ag/AgCl electrode was placed into the left craniotomy. The CFE was implanted through the right craniotomy using a stereotaxic micromanipulator 4.5mm deep into the brain manually.

### RNA Isolation, cDNA and quantitative PCR

Mice were sacrificed using rapid cervical dislocation and brains were rapidly extracted, frozen, and stored at − 80°C for further processing. Microdissected VTA punches were homogenized mechanically using a QIAshredder spin-column (Qiagen). Total RNA was extracted using RNeasy Plus Micro Kits (Qiagen) following the manufacturer’s protocol. gDNA was eliminated prior to extraction with the provided gDNA Eliminator column. NanoDrop 2000 UV-Vis spectrophotometer (Thermo Fisher Scientific) was used to determine concentration and quality of total RNA. cDNA was synthesized from 150 ng of total RNA with iScript cDNA Synthesis Kit (Bio-Rad). cDNA was used to measure gene expression with qPCR. Briefly, sample cDNA (1ng) was loaded with SsoAdvanced Universal SYBR Green Supermix (Bio-Rad) and forward and reverse primers for specific genes of interest. Triplicate samples were amplified on a 96-well plate with the CFX96 Real-Time PCR Detection System (Bio-Rad). Relative gene expression was calculated using the 2^−ΔΔCt^ method, normalized to reference gene Gapdh, and reported as mean ± SEM.

List of primers used in this study:

Gapdh

Forward: AGGTCGGTGTGAACGGATTTG

Reverse: TGTAGACCATGTAGTTGAGGTCA

Gad65

Forward: TCCGGCTTTTGGTCCTTCG

Reverse: ATGCCGCCCGTGAACTTTT

Gad67

Forward: CACAGGTCACCCTCGATTTTT

Reverse: ACCATCCAACGATCTCTCTCATC

### TH

Forward: TGC AGC CCT ACC AAG ATC AAA C

Reverse: CGC TGG ATA CGA GAG GCA TAG TT

### DRD2

Forward: ACCTGTCCTGGTACGATGATG

Reverse: GCATGGCATAGTAGTTGTAGTGG

### DRD3

Forward: CCTCTGAGCCAGATAAGCAGC

Reverse: AGACCGTTGCCAAAGATGATG

### Data Analysis

Each SWV response was first filtered using a zero-phase, forward and reverse (using the filtfilt function on MATLAB), low-pass, third-order Butterworth digital filter with the 3 dB cutoff at a normalized frequency of 0.2 (2 Hz). The fit for the linear baseline was determined using a two-step peak extraction method consisting of an iterative peak localization algorithm. First, a linear baseline was initialized with two signal points on either side of a user-selected peak maximum voltage (~ 0.15 V). Signal points used to construct the baseline were iteratively updated to produce a final baseline that maximized the subtracted peak amplitude. The resulting linear fit intersected boundary points at either side of the DA redox peak profile. The five data points immediately adjacent to the upper and lower bounds were then modeled using linear fitting and subtracted from the raw SWV response for peak extraction.

## Data Availability

The data presented in this study are available on request from the corresponding authors.
